# A study on psychological determinants of users' autonomous vehicles adoption from anthropomorphism and UTAUT perspectives

**DOI:** 10.3389/fpsyg.2022.986800

**Published:** 2022-08-16

**Authors:** Yuqi Tian, Xiaowen Wang

**Affiliations:** ^1^School of Agricultural Economics and Rural Development, Renmin University of China, Beijing, China; ^2^School of Economics, Lanzhou University, Lanzhou, China

**Keywords:** autonomous vehicle, anthropomorphism, user psychology, unified theory of acceptance and use of technology, perceived value theory

## Abstract

As the autonomous vehicles technology gradually enters the public eye, understanding consumers' psychological motivations for accepting autonomous vehicles is critical for the development of autonomous vehicles and society. Previously, researchers have explored the determinants of fully autonomous vehicles but the relevant research is far from enough. Moreover, the relationship between anthropomorphism and users' behavior has been ignored to a large extent. Therefore, this study aim to fill the gap by using anthropomorphism and the unified theory of acceptance and use of technology (UTAUT) to explore how system attributes (i.e., perceived anthropomorphism, perceived intelligence) and UTAUT attributes influence consumers' acceptance behavior. The data were collected *via* questionnaire survey conducted in Beijing, China, which can be a promising early adopter of AVs. Structural equation modeling was used to analyze the data. The results reveal that perceived anthropomorphism and perceived intelligence have a direct positive influence on the adoption of AVs; performance expectancy, effort expectancy, and facilitating conditions have an indirect positive influence on intention to adopt AVs. Also, this research contributes to the literature by enriching studies on psychological determinants of autonomous vehicles' adoption by taking an initial step to highlight anthropomorphism perceptions. This can provide managerial implications for policy-makers and businesses on how to effectively allocate resources to enhance autonomous vehicle adoption.

## Introduction

High technological development (e.g., artificial intelligence, automation technologies) is contributing to the advancement of autonomous vehicles (AVs) which can be an important part of urban transportation systems. Many trails on autonomous cars are being done and AVs are expected to be commercialized by 2040. China's first commercial AVs trail was launched in Beijing in 2021^1^. Around 27 provinces in China have revised AVs regulations and encouraged the development of AVs^2^. In other countries, AVs tests are also being done to test the functions and feasibility (e.g., Google's self-driving car).

Many scholars have confirmed that emergence of AVs can largely influence urban transportation, that's why researchers have done much studies on this topic. One hot sub-topic is the technical design and progress of autonomous technologies (Chen et al., 2017; Zong et al., 2018). These studies are conducted from technology developers' perspectives, trying to develop more reliable technologies and supporting infrastructure.

On the other side, the eventual acceptance of AVs by customers is an imperative trend. Therefore, that means it is equally important to study the psychological feelings of end users' side. We reviewed relevant literature from Scopus and google scholar and found some interesting articles (Adnan et al., 2018; Bernhard et al., 2020; Lajunen and Sullman, 2021; Yuen et al., 2021b, 2022). While there are many studies investigating users' adoption behavior of AVs, the research is far from comprehensive. From the theory and methodology perspective, we found that many research focused on socio-demographic factors (Charness et al., 2018; Park et al., 2021). They examined the heterogeneity of users, such as how users of different ages behave differently. Also, a lot of research focused on psychological motivations using applicable user psychology or technology-relevant theories. Widely applied theories include the unified theory of technology acceptance theory, innovation diffusion theory, trust theory, etc. They examined how technical characteristics (e.g., perceived ease of use), emotional or cognitive characteristics (e.g., emotional response) or social-demographic characteristics (e.g., income) influence the adoption of AVs. However, we noticed quite limited research has focused on how the anthropomorphism of AVs (e.g., audio or visual human likeness) can influence user psychology.

Anthropomorphism is defined as “*the attribution of human characteristics of behavior to any other nonhuman entity in the environment*” (Urquiza-Haas and Kotrschal, 2015). It can be used to describe physical or mental animal-human behavior interaction (e.g., dogs or cats mimic human behavior by showing teeth or smiling). Because humans have a tendency to be more emotionally comfortable with things that are similar to themselves, researchers in psychology field pointed out that anthropomorphism can influence human psychology and behavior (e.g., humans are intimate to dogs that can show human-like comforts) (Lee et al., 2018). With the development of artificial intelligence, technology is displaying anthropomorphism characteristics and interact with humans as well. For example, chatbots and robots are gradually being used in daily life. Some robots are designed in human-like appearance and make human sounds to make people feel comfortable with them. Relevant research found technology that has social attributes (i.e., anthropomorphism) can have influence on user mental statues (Christoforakos and Diefenbach, 2022). Currently, most research on technology orthomorphism is about social robots. AVs receive much less attention from researchers and this is an important research gap.

This research gap should be filled because anthropomorphism is one key service function of artificial intelligence, including AVs. Waytz et al. (2014) found anthropomorphism can increase user trust but we have not completely understood how anthropomorphism influence user adoption intention, which is an interesting and important aspect to explore. Hence, we aim to enrich academic research by considering from combined anthropomorphism, perceived value, and UTAUT perspectives.

Our research question is: What is the relationship between perceived anthropomorphism, perceived intelligence, UTAUT factors, perceived value and users' intention to adopt AVs? We choose UTAUT because it includes more factors compared with technology acceptance model (TAM). TAM explains technology adoption behavior from perceived ease of use and perceived usefulness, but UTAUT has other social factors. Also, UTAUT's effort expectancy and performance expectancy can replace perceived ease of use and perceived usefulness (Li et al., 2021a; Yuen et al., 2021a). We choose perceived value because it is proved to be a good mediator between UTAUT and intention to use; customer value is important in daily businesses. To facilitate city development, we would like to deeply understand the role of perceived value. So, we hypothesis UTAUT factors have a positive effect on the perceived value of AVS; system factors (i.e., perceived anthropomorphism, perceived intelligence) have a positive effect on the perceived value of AVs; perceived value has a positive effect on the intention to adopt AVs. We collected data from Beijing, China through an online questionnaire because of the COVID-19. We analyzed the data using structural equation modeling with AMOS.

## Literature review

### Research scope, model, and theories

The main types of AVs include private AVs, public AVs, and shared AVs. Because each type of AVs has its own characteristics and it is unrealistic to cover all types of AVs in one piece of research, we limit our research scope on private AVs. The reason we choose private AVs is private AVs can be an important factor in smart cities. Moreover, currently, a large number of transport means are private cars. The motivation of the private car drivers can become potential users of AVs and contribute to the sustainable development of society. Hence, it is important to investigate the motivations or resistance to use of private AVs.

AVs are defined in different levels based on the degree of automation^3^. Level 0 is “driving automation” where the driver should be fully responsible for driving. Level 1 is called “driver assistance” where just a single automaton system exits. Level 2 is called “partial driving automation” where steering and broke as well as acceleration support are both automated. Level 3 is called “conditional driving automation” where human drives should override autonomous systems in many cases. Level 4 is called “high driving automation” where human driver override is not necessary in most cases. Level 5 is called “full driving automation” where no human driving is required at all. In this study, we focus on level 5, which is fully autonomous vehicles.

**Figure 1:**
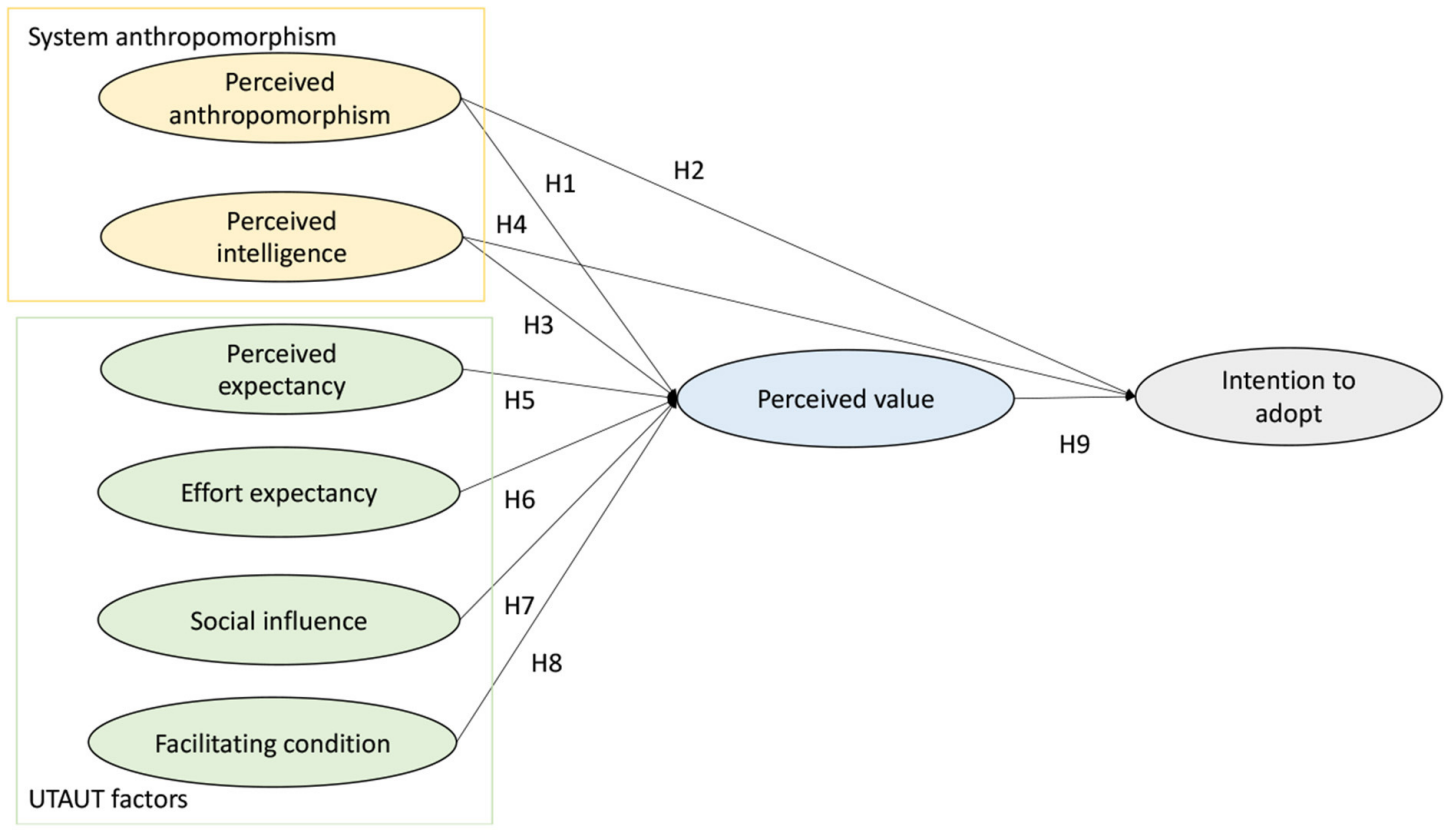
Research framework.

The theoretical framework applied in this research is composed of anthropomorphism theory, Unified Theory of Acceptance and Use of Technology (UTAUT) and perceived value theory. Anthropomorphism is an important concept in psychology. Anthropomorphism refers to non-human agents which have human-like traits, such as actions, sounds, cognitions or emotions. Artificial intelligence has developed to anthropomorphize by developing psychological properties. Designers are also trying to anthropomorphize AVs from both exterior designs and internal systems. They make AVs look friendly and behave more intelligently. Waytz et al. (2014) stated that anthropomorphized technology can make AVs have higher navigation abilities because AVs can be more sensitive to their surroundings. While researchers pointed out anthropomorphism can influence human cognition, the relationship has been rarely studied in AVs field. We would like to find out the underlying relationship between anthropomorphism, customer value and customer intention. Hence, we hypothesis both direct and indirect links. UTAUT is a well-used technology acceptance model to explain users' intention of accepting AVs. Customers' perception on technology characteristics like effort used and benefits, is proven to influence customer attitude, together with social-relevant factors. Perceived value theory describes how consumers' perceived economic, functional, social, environmental or emotional values can affect their decisions on a certain product. Perceived value reflects customers' thoughts and perceptions on the utilities of product. Perceived value theory states that rational consumers will choose to purchase a product that can give them the highest utilities (Payne et al., 2017). Higher perceived value means customers think the product can give their life benefits with a low cost. Many references also proved perceived value can be linked to customer purchase intention (Gounaris et al., 2007; Khoi et al., 2018; Jiang et al., 2021). So we agree it is reasonable to integrate perceived value and customer intention to adopt. Perceived value is also shown to be a strong mediator between UTAUT and customer intention, so we hypothesis indirect relationship between UTAUT and intention to adopt. We link anthropomorphism, technical attributes and consumer utilities together and give a deeper insight into consumers' intention toward AVs. The framework is displayed in Figure 1.

### Research hypothesis

Research on social robots found anthropomorphism has a positive effect on technology perceptions (Jörling et al., 2020). Tong et al. (2020) also pointed that anthropomorphism is a good strategy that can reduce consumers' perceived risks and enhances trust. AVs and social robots share the similarities that they all use artificial intelligence and make human-like decisions. Users may tend to trust AVs functions more when AVs display human traits (Waytz et al., 2014). They may be inclined to believe AVs can really act like humans and make sensible decisions based on real-time conditions. Hence, they perceive AVs can bring them higher value and are willing to adopt AVs. It is a hypothesis that needs to be tested. Hence, from the above discussion, we propose the following hypothesis:

**Hypothesis 1 (H1):** Perceived anthropomorphism has a positive effect on the perceived value of autonomous vehicles.
**Hypothesis 2 (H2):** Perceived anthropomorphism has a positive effect on the intention to adopt autonomous vehicles.

Perceived intelligence is users' perceptions of AVs' abilities to interact with surroundings fluently and naturally. AVs can be considered as intelligent agents that can drive by themselves. It can receive information from sensors and make decisions on speed or direction. If users have high perceptions on the intelligence of AVs system, they tend to believe that AVs can make wise decisions in complex environments and have higher trust in the utilities AVs can provide (Thill et al., 2015). If the users don't perceive AVs systems have high intelligence, they are inclined to doubt AVs' functions and distrust that AVs can bring them high functional or other utilities. When users are convinced AVs systems are intelligent, they are likely to be more willing to use AVs as AVs systems can be intelligent enough to replace human drivers. From the above discussion, we propose the following hypothesis.

**Hypothesis 3 (H3):** Perceived intelligence has a positive effect on the perceived value of autonomous vehicles.
**Hypothesis 4 (H4):** Perceived intelligence has a positive effect on the intention to adopt autonomous vehicles.

Perceived expectancy is the perceived benefits users can obtain from using AVs. Previous research pointed out that AVs can bring usefulness such as improved safety by reducing the accidents arising from human mistakes; AVs can also optimize traffic and reduce congestion by artificially optimized routes (Adnan et al., 2018). Moreover, researchers believe AVs can increase society's sustainability by reducing emissions (Sousa et al., 2018) and encouraging the old generation to self-derive (Maeng and Cho, 2022). When users perceive AVs can give them benefits, they can perceive higher functional, societal and environmental value of AVs. Therefore, we propose the following hypothesis.

**Hypothesis 5 (H5):** Perceived expectancy has a positive effect on perceived value of autonomous vehicles.

Effort expectancy is the ease of driving AVs for users. The degree of ease of use can influence the perceived financial costs of commanding a new skill (Li et al., 2021b). When users feel the AVs' design is user-friendly and easy to understand, they can use less time and costs to learn how to use AVs. Also, the perceived efforts can influence consumers' views on functional utilities as understandable systems can allow users to more efficiently make use of all functions of AVs (Yuen et al., 2022). From the above discussion, we propose the following hypothesis:

**Hypothesis 6 (H6):** Effort expectancy has a positive effect on the perceived value of autonomous vehicles

Social influence is the social norm that can influence users' thoughts and decisions. Humans are a part of society and their behavior can be shaped by the surrounding environments (Panagiotopoulos and Dimitrakopoulos, 2018). If important people of users have positive views and are willing to use it, users can be influenced by important people's opinions and inclined to perceive higher value of AVs. On the contrary, if surrounding people are negative about AVs functions and values, users are inclined to be influenced by their opinions and perceive lower value of AVs. Hence, we propose the following hypothesis.

**Hypothesis 7 (H7):** Social influence has a positive effect on the perceived value of autonomous vehicles.

While facilitating condition is also an external factor as social influence, it is different from social influence as it refers to the available resources that can give users support. The availability of helpful resources can help users to be more confident of using AVs as they can get necessary assistance from the outside. If users have sufficient support, they can make good use of AVs' advantages to travel or live and perceive higher functional utilities. Also, through external support, users can learn how to use AVs at lower costs. If they have enough knowledge of AVs, they can use AVs with fewer efforts and their perceived economic value can be increased. Hence, we propose the following hypothesis:

**Hypothesis 8 (H8):** Facilitating condition has a positive effect on the perceived value of autonomous vehicles.

If AVs can provide high economic utilities such as lower costs, functional utilities such as higher safety and avoidance of human error, environmental functions such as reduced congestion and pollution, or emotional utilities such as fun of driving, users are inclined to be willing to adopt AVs. Past literature has also supported that the perceived value of AVs can influence users' willingness to use AVs (Wang et al., 2019). From the above discussion, we propose the following hypothesis.

**Hypothesis 9 (H9):** Perceived value has a positive effect on the intention to adopt autonomous vehicles.

## Materials and methods

### Survey design

The questionnaire items are displayed in Table 1. The questionnaire items were adapted from previous research.

**Table 1:** Questionnaire items.

**Construct**	**Items**	**References**
Perceived intelligence	Autonomous vehicles are competent in providing driving services	(Bartneck et al., 2009; Balakrishnan et al., 2022)
	Autonomous vehicles exhibit responsibility during driving	
	Autonomous vehicles are knowledgeable during driving	
Perceived anthropomorphism	Autonomous vehicles can be humanlike	(Bartneck et al., 2009; Balakrishnan et al., 2022)
	Autonomous vehicles can be conscious of their actions	
	Autonomous vehicles can be elegant in engaging	
Performance expectancy	I will find autonomous vehicles useful in my daily life	(Madigan et al., 2017)
	Using autonomous vehicles can increase my productivity	
	Using autonomous vehicles can help me accomplish things more quickly	
Effort expectancy	My interaction with autonomous vehicles would be clear and undrainable	(Madigan et al., 2017)
	I would find autonomous vehicles easy to use	
Social influence	People who are important to me think that I should autonomous vehicle	(Madigan et al., 2017)
	People who influence my behavior think that I should use autonomous vehicle	
	People whose opinions I value would like me to use autonomous vehicle	
Facilitating conditions	I would have the resources necessary to use APT	(Madigan et al., 2017)
	I would have the knowledge necessary to use APT	
	I would be able to get help from others when I have difficulties using APT	
Perceived value	I feel that using autonomous vehicles can better cater to my travel requirements (e.g., safety, reliability, security or convenience needs)	(Boksberger and Melsen, 2011; Yuen et al., 2021c)
	I feel that using autonomous vehicles can provide economic benefits	
	I feel that using autonomous vehicles can cater to my emotional needs	
	I feel that using autonomous vehicles would have positive effects on the environment and society	
Intention to adopt	I would recommend autonomous vehicles to my family and peers	
	I would encourage others to use autonomous vehicles	
	I would consider using autonomous vehicles when they are available in the market	

The questionnaire is designed in three sections. In the first section, we introduce the purpose of the survey and the scope (e.g., focus on fully autonomous vehicles) to make participants clear about the background. In the second section, we list all questionnaire questions as shown in Table 2. Seven-point Likert scale is used. Participants are asked to rate from “1 = strongly disagree” to “7 = strongly agree.” Attention checker is also mixed in this section. The attention checker asked respondents to select a defined answer (i.e., please select the third answer for this question). In the third section, we ask participants to submit their demographic information.

**Table 2:** Demographic information.

**Demographic information**		**Frequency**	**Percentage %**
Gender	Male	161	51
	Female	154	49
Age	19–30	121	38
	31–40	108	34
	41–50	56	19
	51–60	20	6
	>61	10	3
Car owner	Yes	254	80
	No	61	20

The data were collected *via* online questionnaire surveys in Chinese. Because COVID-19 can limit the distribution of offline questionnaires, online questionnaire is a safe way to collect data. The surveys were administrated with the assistance of Wenjuanxing, a professional online survey company. A reward equaling $2 was given to participants who completed the questionnaire. We collected data from March 1st to March 30th 2022. In total, we distributed 950 questionnaires. Finally, we collected 354 questionnaires. We deleted the invalid questionnaires. The invalid questionnaires are those that failed to answer attention checker questions. Failing to answer the question indicates that the respondents didn't read instructions carefully and their responses cannot be trusted. Finally, we obtained 315 valid responses.

### Non-response bias and common method bias

Non-response can be a trouble for self-administrated surveys. We used *t*-test to examine non-response bias. The data are split into two parts based on the completion time and the result shows no significant difference between the two groups (Armstrong and Overton, 1977). Hence, we conclude non-response bias is not a problem in our research.

Harman's single factor is used to examine common method bias. The total variance of the single factor model is 37.5% and the value is smaller than the cut-off value of 50% (Podsakoff et al., 2003). Hence, we conclude common method bias is not a concern.

## Results and discussion

### Demographic information

The demographic information is displayed in Table 2. As shown in Table 2, 49% of the respondents are female and 51% are male. In 2020, the ratio of male and female residents in Beijing is 51.15 and 48.66%, respectively^4^. The ratio of the whole population is similar to the respondents in this survey. From the age distribution, we know the majority of respondents are below 50 years old. Thirty-eight percentage of the respondents are below 30 years old. Hence, the respondents are relatively young. Moreover, 80% of the respondents are current private car owners and familiar with car operations.

### Confirmatory factor analysis

Before doing structural equation modeling, we did confirmatory factor analysis to examine the structural reliability and validity of the proposed model. As shown in Table 3, the model fits are satisfactory [the comparative fit index (CFI) and Tucker-Lewis fit index (TLI) are above 0.90; the root mean square error of approximate (RMSEA) is <0.08; the standardized root mean square residual (SRMR) is <0.10 (Hu and Bentler, 1999)].

**Table 3:** Confirmatory factor analysis results.

**Construct**	**Indicator**	**λ**	**AVE**	**CR**
Perceived anthropomorphism (PE)	PA1	0.865	0.828	0.935
	PA2	0.953		
	PA3	0.911		
Effort expectancy (EF)	EE1	0.844	0.738	0.849
	EE2	0.874		
Facilitating conditions (FA)	FC1	0.889	0.782	0.917
	FC2	0.949		
	FC3	0.819		
Social infleucne (SO)	SI1	0.762	0.551	0.786
	SI2	0.775		
	SI3	0.687		
Perceived intelligence (PI)	PI1	0.881	0.708	0.879
	PI2	0.852		
	PI3	0.790		
Performance expextancy (PR)	PE1	0.835	0.702	0.876
	PE2	0.849		
	PE3	0.830		
Perceived value (PV)	PV1	0.829	0.668	0.889
	PV2	0.879		
	PV3	0.756		
	PV4	0.801		
Adoption (AD)	AD1	0.833	0.663	0.855
	AD2	0.780		
	AD3	0.829		

*Model fit indices: χ^2^/df = 2.85, (p < 0.001); CFI = 0.954; TLI = 0.945; RMSEA = 0.063; SRMR = 0.054*.

Composite reliability (CR) and average variance extracted (AVE) values are symbols of model reliability. As shown in Table 3, the AVE values are higher than 0.5 and CR values are higher than 0.80 (Hair et al., 2010). Hence, the structural reliability is conformed.

Moreover, the square root of AVE values are higher than the correlations between constructions. We provide the numbers in Table 4. Hence, the structure's discriminant validity is confirmed. We can proceed to do structural equation modeling to test hypotheses.

**Table 4:** Discriminant validity.

	**PE**	**PI**	**AD**	**PV**	**EF**	**PR**	**FA**	**SO**
PE	0.910	0.853	0.327	0.361	0.153	0.161	0.177	0.210
PI		0.841	0.385	0.360	0.139	0.180	0.133	0.267
AD			0.814	0.625	0.563	0.574	0.474	0.795
PV				0.817	0.565	0.666	0.635	0.555
EF					0.859	0.784	0.688	0.746
PR						0.837	0.732	0.647
FA							0.884	0.632
SO								0.742

**Table 5:** Structural equation modeling results.

**Hypotheses**	**Estimate**	***P***	**Conclusion**
Perceived anthropomorphism → Perceived value	0.165	0.076	Not supported
Perceived intelligence → Perceived value	0.109	0.149	Not supported
Performance expectancy → Perceived value	0.355	0.000**	Supported
Effort expectancy → Perceived value	0.152	0.000**	Supported
Social influence → Perceived value	−0.101	0.286	Not supported
Facilitating conditions → Perceived value	0.283	0.000***	Supported
Perceived anthropomorphism → Intention to adopt	0.278	0.050*	Supported
Perceived intelligence → Intention to adopt	0.296	0.000***	Supported
Perceived value → Intention to adopt	0.358	0.000***	Supported

### Structural equation modeling

We tested the direct relationship between UTAUT factors and intention to use. The relationships are not significant. So we conclude in our survey, perceived value is a full mediator. Direct relationship between UTAUT and customer intention is not significantly supported. We conclude our model is reasonable and robust.

The model fits of structural equation modeling are displayed in Table 4. The research model has good model fits (χ^2^*/df* = 2.96; CFI = 0.934; TLI = 0.916; RMSEA = 0.07; SRMR = 0.06). The R square of perceived value is 0.623 and that of intention of adopt AVs is 0.745.

The performance expectancy, effort expectancy and facilitating conditions are shown to have positive and significant effects on the perceived value of AVs. The results supplement empirical evidence to the influence of UTAUT factors on consumer adoption of AVs (Kettles and Van Belle, 2019; Smyth et al., 2021). Performance expectancy has a positive effect on perceived value. The result indicates that the advantages of AVs should be fully exploited to improve their values of AVs. For example, the safety of AVs should be ensured; the AVs should provide high quality service to users. In terms of effort efficacy, the interaction between AVs and users should be made user-friendly so that users can spend less time and cost learning how to use AVs. For example, the commanders can be made straightforward to users and the functions can be made similar to traditional cars. Regarding facilitating conditions, the result supports that necessary resources should be provided to users. Surprisingly, with regards to social influence, the result suggests that the effect is not significant. This is contrary to some past research (Zhang et al., 2020). One possible explanation is the respondents of this survey are not easily influenced by others. They have strong self-judgment and are more willing to believe in themselves. Therefore, available supportive resources and belief in AVs' performance can increase their perceived value but social influence does not exhibit significant influence. This reflects the special characteristics of the residents of the capital of China, a more developed city. Surveyed people are from developed cities and receive good education. It is possible they have ability to judge by themselves and so, are less easily persuaded by other people. They may think following the crowd is not wise and they like to control their own thoughts and behavior. The result implies business partners should be careful with using different advertisements strategies according to their targeted customers.

Perceived intelligence and perceived anthropomorphism are shown to directly positively influence the adoption of AVs. However, the direct influence from perceived anthropomorphism and perceived intelligence on perceived value is not significant. The result empirically supports those perceptions of anthropomorphism can influence user perceptions and behavior. The result also implies that human-machine interaction is associated with users' willingness to adopt AVs. If users are convinced AVs act like humans and have high intelligence and are trustworthy, they tend to trust AVs can improve their life by making driving safer and cheaper and are willing to adopt AVs. However, perceived value is not shown to be a significant mediator between system factors and the adoption of AVs. A possible explanation is that system factors are more associated with trust of AVs (Chan and Lee, 2021) while UTAUT factors are more associated with the perceived value of AVs. In the future, researchers can explore other mediators to more deeply investigate the relationship between system factors and adoption of AVs. The empirical result of this research suggests designers and developers should optimize the intelligent system of AVs, making AVs safe, trustworthy and easy to be adopted by users. However, some researchers proposed uncanny valley effects which describe a “non-linear relationship between anthropomorphism and affinity” (Złotowski et al., 2015). While our research takes an initial attempt to reveal a positive relationship between perceived anthropomorphism and perceived value, the non-linear relationship cannot be discussed and is worth being more deeply studied.

Perceived value is shown to positively influence users' willingness to adopt AVs. The result is consistent with the research done by Yuen et al. (2020). The result is also similar to research on the adoption of electric vehicles (Kim et al., 2018). Increasing AVs' economic, functional, societal, and environmental values is necessary for transforming the public's attitude toward AVs.

## Conclusions

Many countries are investing heavily in the development of autonomous vehicles and expect to transform society in the coming years (or gain a foothold in the AVs market). Consumers' perceptions of AVs should be clearly understood to adapt to this transformation is imminent. Our research aims to enrich the relevant research by investigating the relationship between perceived anthropomorphism, perceived intelligence, UTAUT factors, perceived value, and intention to adopt AVs. The novelty of our research is our model goes beyond UTAUT factors which have been discussed in past studies and combines anthropomorphism theory to investigate how perceptions on anthropomorphism and intelligent systems can together influence user psychology. Anthropomorphism is one key characteristic of artificial intelligence but has been less discussed in research on users' adoption psychology and behavior of AVs. The research findings imply that perceived anthropomorphism, and perceived intelligence have a direct positive effect on the intention to adopt AVs and three UTAUT factors (i.e., performance expectancy, effort expectancy, facilitating conditions) have a positive indirect significant influence on the intention to adopt AVs *via* perceived value.

## Theoretical contribution

Our research makes contributions in the following dimensions. The first dimension is we propose a model combining users' perceptions of anthropomorphism. Investigation into relationship between anthropomorphism and user behavior is important for future development of AVs. The result enrich research on user psychology and user behavior by providing more advanced perspectives.

## Practical implication

Also, our research findings provide implications for designers and policy-makers. First, they should maximize the utilities AVs can bring to consumers. They should optimize the safety and reliability of AVs systems and make the AVs more cost-effective (e.g., fuel-efficient). Second, they should simplify the design to make it more user-friendly. As old generations and the disabled can be a big potential market segment, designers can continue to collect data on potential users' needs and upgrade their designs based on received feedback. Third, necessary infrastructure and supporting learning resources should be provided to provide facilitating conditions. The benefits of AVs can be circulated to the public to enhance belief in the reliability of AVs. From the findings on the positive relationship between perceived anthropomorphism and perceived intelligence, we suggest designers put efforts into the development of intelligent systems. They can make AVs intelligent, responsive, and trustworthy. Simple anthropomorphic design elements include the exterior design of AVs (e.g., make it human-like); other elements include the sound and visual quality. To enhance system intelligence, governments can provide support on human/financial resources as well.

## Limitation and recommendations

Our research has several limitations. The first limitation is that the survey was only conducted in one metapolitician of China. Because of time and financial cost limitations, it is rather difficult to do surveys in other cities/countries by the authors alone. Although the authors have tried to make sure the respondents are representative, the results can be not easy to be generalized to other regions because of cultural differences. Now, we are trying to collect data from less developed cities, like cities in the western of China, to compare results and find differences. The second limitation is that we adapt factors from anthropomorphism, UATUT and perceived value theories. The results suggest that the model has good explanatory power. But more phycological and cognitive factors remain unexplored. Thus, we suggest future researchers can explore more appropriate factors and theories to understand the psychological motivations of autonomous vehicle acceptance. The third limitation is that we did not exhaustively explore the heterogeneity of the results. Thus, we suggest that researchers can use ANOVA or other methods (e.g., control variable method) to understand the more deeply about the heterogeneity. The fourth limitation is that we could not investigate deeper into the anthropomorphism concept. From our research findings which links the anthropomorphism and user utilities, researchers can further investigate the change of utilities in different degree of anthropomorphism. Researchers can also investigate consumers' reactions to different

## Data availability statement

The original contributions presented in the study are included in the article/supplementary material, further inquiries can be directed to the corresponding author.

## Ethics statement

The studies involving human participants were reviewed and approved by Tianjin Vocational Institute, China. The patients/participants provided their written informed consent to participate in this study. The study was conducted in accordance with the Declaration of Helsinki.

## Author contributions

YT has conceptualized the concept, collected data, analyzed the data, and wrote the draft. XW has conceptualized the idea, supervised, and wrote the draft. Both authors contributed to the article and approved the submitted version.

## Conflict of interest

The authors declare that the research was conducted in the absence of any commercial or financial relationships that could be construed as a potential conflict of interest.

## Publisher's note

All claims expressed in this article are solely those of the authors and do not necessarily represent those of their affiliated organizations, or those of the publisher, the editors and the reviewers. Any product that may be evaluated in this article, or claim that may be made by its manufacturer, is not guaranteed or endorsed by the publisher.

## Footnotes

^1^ https://www.globaltimes.cn/page/202111/1239891.shtml

^2^ http://www.news.cn/english/2021-12/09/c_1310361852.htm

^3^ https://www.synopsys.com/automotive/autonomous-driving-levels.html

^4^ http://tjj.beijing.gov.cn/
